# Hydroxyapatite From the Skull of Tuna (*Thunnus obesus*) Head Combined With Chitosan to Restore Locomotive Function After Spinal Cord Injury

**DOI:** 10.3389/fnut.2021.734498

**Published:** 2021-08-23

**Authors:** Chen-chen Ma, Xi-chang Wang, Ning-ping Tao

**Affiliations:** ^1^College of Food Science and Technology, Shanghai Ocean University, Shanghai, China; ^2^Shanghai Engineering Research Center of Aquatic-Product Processing and Preservation, Shanghai, China

**Keywords:** hydroxyapatite, chitosan, spinal cord injury, tuna head, polymers, nanoparticles

## Abstract

Hydroxyapatite is an important fish bone calcium in tuna head, which is widely used to repair of bone defect. Chitosan is a degradable basic polysaccharide with good biocompatibility and bone guiding, which can achieve targeted delivery to the injured spinal cord after spinal cord injury (SCI). This study aimed to evaluate the beneficial effects of chitosan combined hydroxyapatite (chitosan-hydroxyapatite) nanoparticles on SCI. The result revealed the chitosan-hydroxyapatite particles were successfully constructed and the stability of particles was maintained at low temperature. Moreover, we found chitosan-hydroxyapatite administration could improve SCI, while chitosan alone treatment resulted in no significant increase of the Basso Beattie Bresnahan (BBB) scores compared with the control group. In addition, chitosan-hydroxyapatite particles also significantly reduced the lesion cavity volume and improved the dispersed structure, indicating it could promote the recovery of tissue function of SCI rats. This study explored the effects of chitosan-hydroxyapatite nanoparticles on the location and function of spinal cord injury, provided experimental evidence for further research on its application in spinal cord repair, and helped improve the efficient use of tuna heads.

## Introduction

Tuna is a large, medium-sized and upper-layered fish that is distributed in the middle and low latitudes of the ocean, and has high economic value (1). Tuna is a warm-water migratory fish in the ocean and is one of the three nutritional fish recommended by the International Nutrition Society. In recent years, owing to the rich in high-quality protein, docosahexaenoic acid, eicosapentaenoic acid and other polyunsaturated fatty acids, and the meat is tender and soft, tuna is favored by consumers and is an important ocean food commodity fish (2). Tuna meat is delicious, nutritious, and rich in biologically active substances such as EPA and DHA, but it will produce a lot of scraps, including fish head, offal, fish bone, fish tail and fish skin (3, 4). Tuna head is an important part of the scraps and rich in crude fats and fatty acids, but the large proportion of fish bones in the fish head makes it difficult to process and use (5). Tuna head is boiled to get tuna head soup, which contains a large amount of saturated fatty acids, monounsaturated fatty acids, polyunsaturated fatty acids, and vitamin E, preventing blood lipids, cardiovascular disease such as arteriosclerosis, and can balance the deficiency of n-3 polyunsaturated fatty acids (6, 7). In addition, tuna head soup is rich in various proteins such as collagen, oligopeptides and chondroitin, and has a very rich calcium content, as high as 20–30% (8).

Tuna head soup is a new marine source for the separation of hydroxyapatite (9). Hydroxyapatite is a bone bionic material with good biocompatibility and bone guiding effect, which can promote early osseointegration and bone repair (10, 11). Nano-hydroxyapatite has been reported to have superior bioresorbtion and close chemical and crystallographic structure to ordinary hydroxyapatite (12). Simultaneously, nano-hydroxyapatite has less cytotoxicity and better biological properties. Besides, it is more biocompatible and has a beneficial effect on bone formation (13). The application of nano-hydroxyapatite in stomatology mainly involves many fields such as jaw defect repair, implantation, root canal filling, and dental remineralization (14). Spinal cord injury (SCI) is a devastating neurological disorder that causes significant physiological changes in patients, such as the cardiovascular responses, metabolic pathways and thermoregulation (15). In recent years, with the continuous deepening of research on spinal cord injury, the understanding of spinal cord regeneration and repair after spinal cord injury has been deepened, and the development of cell biology and molecular biology has provided new therapeutic methods for the treatment of SCI (16, 17). However, various methods are not satisfactory for the treatment of spinal cord injury. Therefore, in this study, hydroxyapatite previously extracted from the tuna head was applied to the treatment of SCI. At present, it is the focus of research to further improve the performance of hydroxyapatite so as to improve its efficiency, and one of the methods is to modify its surface. On the other hand, chitosan is a degradable basic polysaccharide with a six-membered ring stable structure, which is suitable for the preparation of stressed materials, and has good biocompatibility and bone guiding (18). Chitosan nanomaterials have become the main scaffolding materials for bone repair, cartilage regeneration, and soft tissue repair due to their good biocompatibility, good mechanical properties, and excellent degradation performance (19). However, the effect of hydroxyapatite combined chitosan nanoparticles on the repair of spinal cord injury remains unknown. In this study, hydroxyapatite nanoparticles were modified by chitosan, which could improve the delivery efficiency by changing the hydrophobicity, surface charge and stability. Also, this study explored the effects of chitosan-hydroxyapatite nanoparticles on the location and function of spinal cord injury, and provided experimental evidence for further research on its application in spinal cord repair.

## Result

### Characteristics of Chitosan-Hydroxyapatite Nanoparticles

To determine the stability and surface charge of chitosan-hydroxyapatite nanoparticles, the particles were incubated at 4°C for 4 weeks. The sizes of chitosan-hydroxyapatite nanoparticles were around 105 nm and the zeta potential of chitosan-hydroxyapatite nanoparticles was found to be nearly 9 mV, which suggested that the stability of the particles was successfully maintained at low temperature for 4 weeks (Table 1). In addition, the sizes of chitosan nanoparticles were around 90 nm and their zeta potential were nearly 11 mV at 4°C in 4 weeks (Table 1).

**Table 1:** Sizes and zeta potentials of chitosan and chitosan-hydroxyapatite nanoparticles (*n* = 3).

**Particles/weeks**	**DLS hydrodynamic (nm)**				
	**0**	**1**	**2**	**3**	**4**
Chitosan	92 ± 3.8	94 ± 4.3	88 ± 5.8	89 ± 3.6	87 ± 4.7
Chitosan-hydroxyapatite	108 ± 6.3	104 ± 5.2	111 ± 3.6	109 ± 4.7	99 ± 4.2
**Particles/weeks**	**Zeta potential (mV)**				
	**0**	**1**	**2**	**3**	**4**
Chitosan	+11.1 ± 1.2	+11.8 ± 0.9	+10.7± 0.8	+11.5 ± 1.6	+10.3 ± 1.2
Chitosan-hydroxyapatite	+9 ± 1.3	+9.2 ± 2.1	+9.7 ± 1.7	+8.8 ± 1.5	+9.5 ± 1.1

### Identification of Chitosan-Hydroxyapatite Nanoparticles

**Figure 1:**
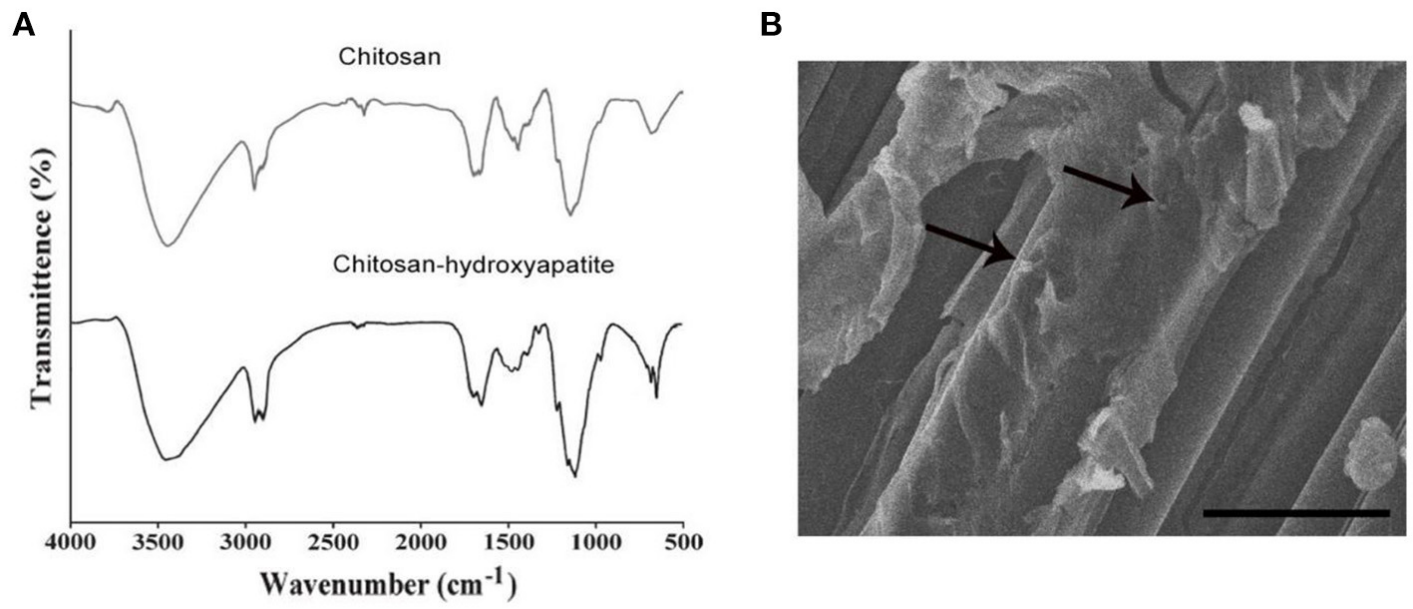
Characteristics and microscopic image of components used for chitosan-hydroxyapatite preparation. **(A)** Infrared spectroscopy images of chitosan scaffold and chitosan-hydroxyapatite. **(B)** Scanning electron micrographs of chitosan-hydroxyapatite. Arrows represent the particles. Scale bar: 100 μm.

The modification of chitosan-hydroxyapatite nanoparticles was confirmed by FTIR analysis. As shown in Figure 1A, hydroxyapatite was successful attached to chitosan nanoparticles. For chitosan-hydroxyapatite nanoparticles, the spectrum showed characteristic absorption of peak around 3,500 cm^−1^ which was attributed to the combined stretching vibration of OH and N-H groups, whereas at 1,700 cm^−1^ was the characteristic peaks of amide. Moreover, C-H stretching of rock was observed at 1,400 cm^−1^, and C-O-C stretching mode at 1,150 and 1,050 cm^−1^. The spectra of peak around 1,070 and 570 cm^−1^ represented ${\text{PO}}_{4}^{3-}$ stretching group, and the peak of 1,450 cm^−1^ indicated the ${\text{CO}}_{3}^{2-}$ stretching vibration, which were the characteristic absorption bands of hydroxyapatite, demonstrating that hydroxyapatite was successfully coated in chitosan nanoparticles. Low-molecular weight chitosan-hydroxyapatite nanoparticles were studied under SEM for the integrity (Figure 1B). It was observed that hydroxyapatite appeared to have a mixture of fine micro and nano granules that were integrated with the chitosan scaffold (Figure 1B).

### Targeted Delivery of Chitosan-Hydroxyapatite to the Injured Spinal Cord

**Figure 2:**
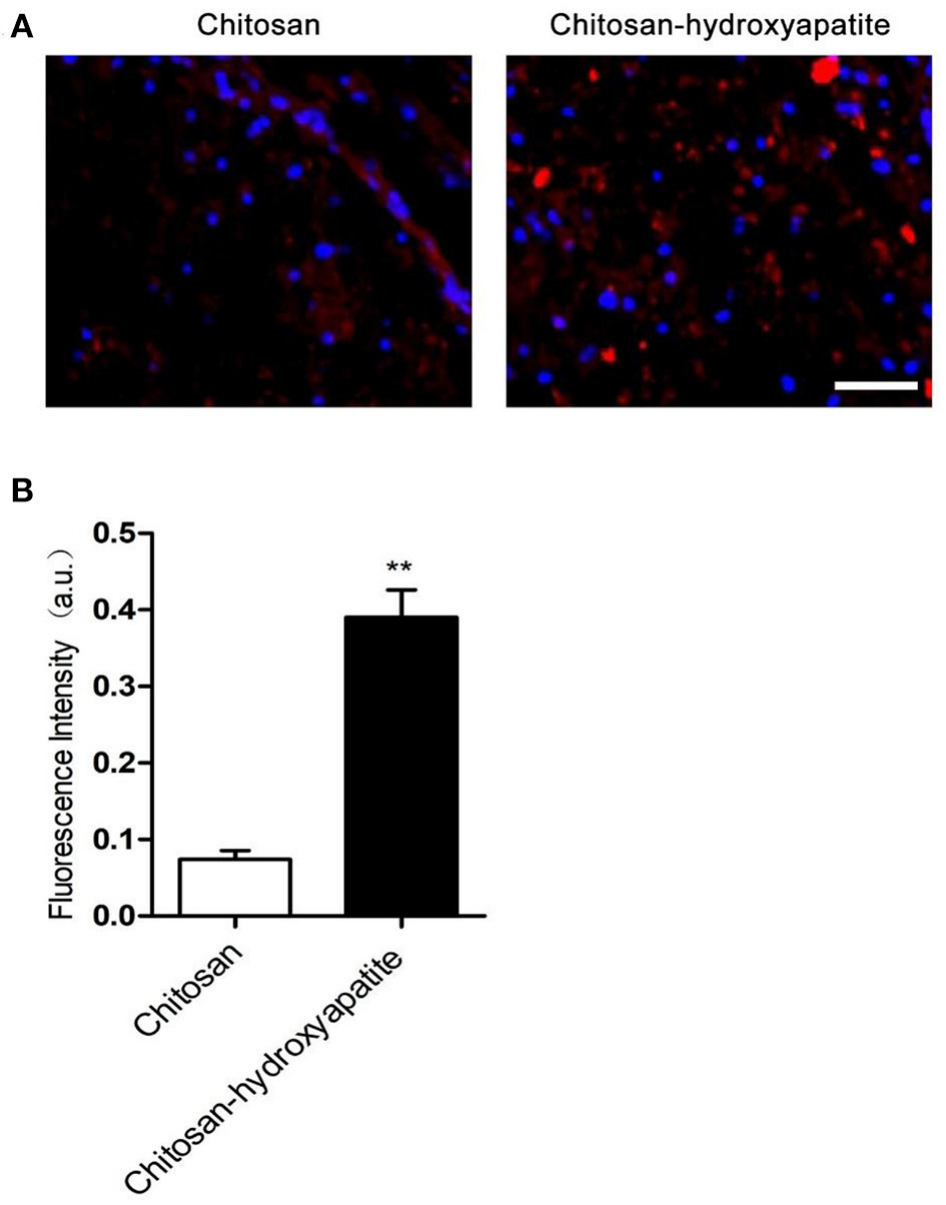
Targeted delivery of chitosan-hydroxyapatite to the injured spinal cord. **(A)** Fluorescence images of chitosan-Cy5.5 and chitosan-hydroxyapatite-Cy5.5 in the injured spinal cord. Red represents Cy5.5, blue represents dapi. Scale bar: 100 μm. **(B)** Quantitative results of fluorescence intensity of Cy5.5 in the labeled chitosan and chitosan-hydroxyapatite (*n* = 4). **P < 0.01.

To investigate the effect of chitosan-hydroxyapatite nanoparticles on targeted delivery to the injured spinal cord, the Cy5.5 was labeled to chitosan-hydroxyapatite nanoparticles at room temperature. The Cy5.5 labeled chitosan-hydroxyapatite and chitosan were used to treat the rats of SCI by intravenous administration and quantified in the injured spinal cord at 48 h after SCI (Figure 2A). The concentration of the particles was measured by detecting the fluorescence intensity of Cy5.5. The result demonstrated that the efficiency of delivery to the injured spinal cord were significantly enhanced by the treatment of chitosan-hydroxyapatite compared with chitosan treatment group, which was estimated through the fluorescence intensity of Cy5.5 at the injured spinal cord (Figure 2B).

### Effect of Chitosan-Hydroxyapatite on the Function Recovery After SCI

**Figure 3:**
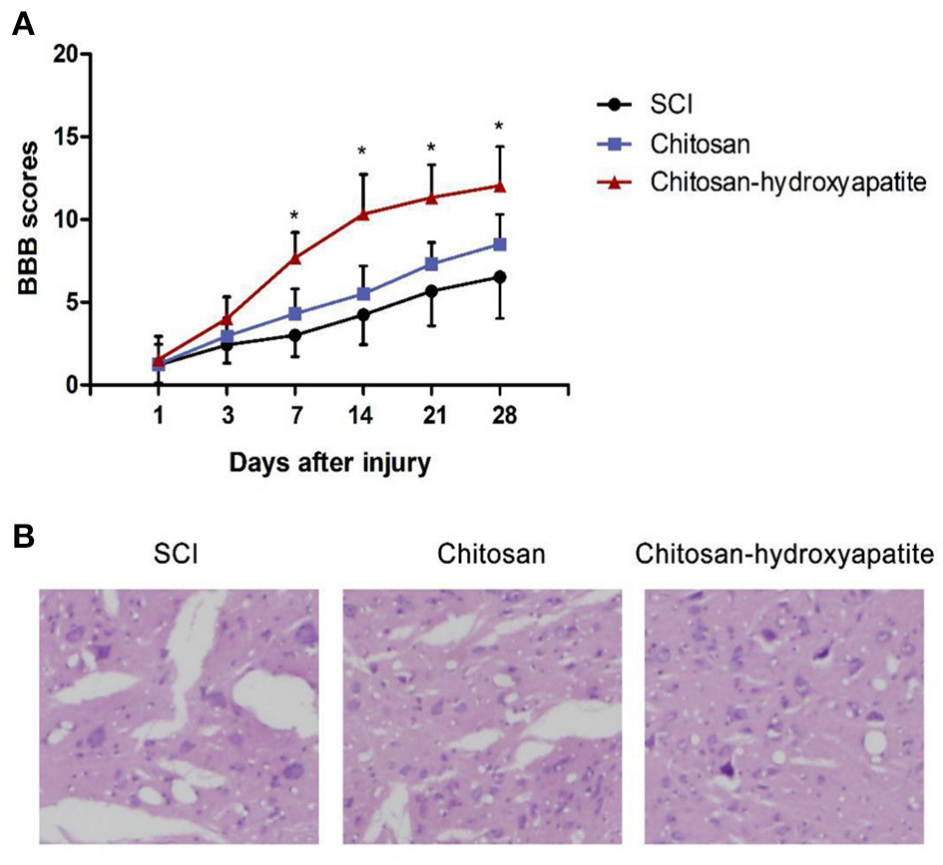
Effect of chitosan-hydroxyapatite on the recovery of spinal cord injury in rats. **(A)** Basso, Beattie and Bresnahan (BBB) scores were evaluated at different time points after injury. *P < 0.05 vs. SCI group. **(B)** The HE staining was performed at 28 days after injury.

To investigate the effect of chitosan-hydroxyapatite on SCI, we assessed the function repair by treatment of chitosan-hydroxyapatite after SCI. The BBB scores were significantly increased at 7 days after treatment of chitosan-hydroxyapatite, compared with the control SCI group (Figure 3A). On the other hand, chitosan alone treatment resulted in no significant increase of the BBB scores at different time points compared with the control group (Figure 3A). In order to explore the effect of chitosan-hydroxyapatite on tissue recovery after SCI, the HE staining was performed at 28 days after injury. The result demonstrated that administration of chitosan-hydroxyapatite significantly reduced the lesion cavity volume, and chitosan treatment resulted in no significant improvement of the lesion cavity volume compared the SCI group (Figure 3B). In addition, the dispersed structure was apparently improved by the chitosan-hydroxyapatite treatment when compared with the SCI and CN treatment group (Figure 3B).

## Discussions and Conclusions

Tuna heads are rich in fish bone calcium. Preparation of fish bone calcium into nano-hydroxyapatite plays an important role in the effective use of tuna head. Nano-hydroxyapatite is widely used in bone injury diseases. It is estimated that there are more than 3 million patients with bone defects caused by traffic, production accidents and trauma, tumors and tuberculosis each year (20). The most commonly used treatment method is allogeneic tissue transplantation for trauma to the bone and severe soft tissue damage (21). However, there are many complications such as infection, delayed or non-union of bone healing, bleeding, and chronic pain, with the risks of immune rejection and disease transmission (22). Owing to the problems with allogeneic tissue transplantation, it is urgent to explore the new treatment methods in trauma surgery. SCI is easily caused by crushing, fracturing, pulling or sharps, bullets, etc., and is difficult to repair. SCI may lead to permanent disability, which places a greater burden on patients and society.

In recent years, with the continuous deepening of research on chitosan nanoparticles materials, it has been found that chitosan nanoparticles materials have good biocompatibility, degradability and osteoinductivity (23). Therefore, researchers have tried to treat SCI by using composite chitosan nanoparticles materials (24). Here, in this study, through biochemical methods, chitosan-hydroxyapatite nanoparticles were formed and evaluated using different manners. The size and zeta potential of chitosan-hydroxyapatite nanoparticles did not change significantly after being stored at 4°C for 4 weeks, which suggested that the stability of the particles was successfully maintained at low temperature. The modification of chitosan-hydroxyapatite nanoparticles was confirmed by FTIR analysis and the integrity of chitosan-hydroxyapatite nanoparticles was testified using SEM.

Previous studies have shown that chitosan nanoparticles can be delivered to the injured spinal cord in a targeted manner (25, 26). To investigate the effect of chitosan-hydroxyapatite nanoparticles on targeted delivery to the injured spinal cord, the Cy5.5 was labeled to chitosan-hydroxyapatite nanoparticles at room temperature. The efficiency of delivery to the injured spinal cord were significantly enhanced by the treatment of chitosan-hydroxyapatite compared with chitosan treatment group through quantification of the fluorescence intensity of Cy5.5 at the injured spinal cord, suggesting that chitosan-hydroxyapatite targeted to the injured spinal cord. Previous studies reported that chitosan nanoparticles might slightly improve the function recovery after SCI (27, 28). Here, in this study, we found that chitosan alone treatment resulted in no significant increase of the BBB scores at different time points compared with the control group. On the other hand, the BBB scores were significantly increased at 7 days after treatment of chitosan-hydroxyapatite when compared with the control SCI group, which suggested that chitosan-hydroxyapatite could promote the function recovery after SCI. In addition, chitosan-hydroxyapatite was also testified to promote the tissue recovery after SCI. In conclusion, this study explored the effects of chitosan-hydroxyapatite nanoparticles on the location and function of spinal cord injury, and provided experimental evidence for further research on its application in spinal cord repair.

## Materials and Methods

### Preparation of Chitosan and Chitosan-Hydroxyapatite Nanoparticles

Hydroxyapatite was prepared and characterized by Fourier Transform infrared spectroscopy (FT-IR) according to the reported methods (9). A total of 2.5 g of low-molecular weight chitosan (1,526 Da) was dissolved in 250 mL of 2% acetic acid solution. The solution was stirred overnight on a mechanical stirrer (RW 20.n Labortechik) and sonicated for 1 h to remove any air bubbles. The solution was lyophilized in freeze dryer to form chitosan scaffolds. A total of 2.5 g of prepared hydroxyapatite was suspended in 50 mL of water and carefully transferred into the chitosan solution. The solution was mechanically stirred for 24 h to disperse the hydroxyapatite particles in the polymer matrix in a homogeneous manner. The mixed solution was lyophilized in freeze dryer to form chitosan-hydroxyapatite nanoparticles.

### Characterization of Chitosan-Hydroxyapatite Nanoparticles

Chitosan-hydroxyapatite nanoparticles samples were prepared by drop drying a water diluted suspension. The mean diameter of the studied samples was determined by statistical analysis. The surface charges of nanoparticles in distilled water were determined using a zeta potential analyzer (the measurement temperature at 25°C), (Brookhaven Instrument Co., CA). The nanoparticles size was characterized through dynamic light scattering (He-Ne laser, wavelength 633 nm, incident Angle 90°, set the measurement temperature at 25°C, time for 5 min, the nanoparticles were placed in the particle size pool Appropriate amount of nanoparticles in the particle size pool) (DLS).

### Fourier Transform Infrared Spectroscopic Analysis

FTIR was used to evaluate the modification of chitosan-hydroxyapatite nanoparticles. The chitosan-hydroxyapatite nanoparticles were recovered by freeze-drying and characterized by FTIR spectroscopic analysis. Briefly, 500 μg nanoparticle powder was mixed and ground with spectroscopic-grade KBr. IR spectra was recorded using an FTIR spectrophotometer (Thermo, USA) within the range of 500–4,000 cm^−1^. Chitosan nanoparticles were determined as control.

### Scanning Electron Microscopy

The structural integrity of chitosan-hydroxyapatite was analyzed and the image was detected by Scanning Electron Microscopy (SEM, HITACHI, Japan). Chitosan-hydroxyapatite and chitosan were fixed overnight in the buffer containing 25 g/L glutaraldehyde (glutaraldehyde was dissolved in 0.1 mol/L sodium diarsonic acid buffer), respectively, and dehydrated with gradient ethanol. The treated samples were freezed for 2 d and Metal spraying.

### Cy5.5-labeled chitosan-hydroxyapatite nanoparticles

Cy5.5 was dissolved in DMSO, added to chitosan or chitosan-hydroxyapatite solution and left at room temperature in the dark for 6 h. The solution was performed with dialysis against distilled water. The amounts of Cy5.5 in the chitosan or chitosan-hydroxyapatite treatment in the injured spinal cord were determined by fluorescence.

### Animals

Adult wistar-rats (males) are 6 weeks old [(180–220 g), Sprague-Dawley, Harlan] were provided by the Animal Center of Second Military Medical University. All of the animals were treated humanely and with regard for the alleviation of suffering. This study was carried out in accordance with the guidelines of the Care and Use of Laboratory Animals of the National Institutes of Health. All experimental protocols described in this study were approved by the Ethics Review Committee for Animal Experimentation of the Second Military Medical University. The permission number for animal experiment is SHOU-DW-2021-074.

### Animal Model of SCI

Allen's method was used to make SCI rat model. The rats were anesthetized with 4% isoflurane. A laminectomy was performed at the thoracic vertebra level 10 (T10) after shaving and cleaning until fully recovered from the anesthesia. Spinal cord contusion was induced using a weight-drop apparatus, where a guided 5 g rod was dropped from a height of 80 mm onto the exposed cord, representing moderate SCI. After surgery, the muscles were sutured in layers and the skin incision was closed with silk threads. Penicillin G (40,000 U, i.m.) was administrated daily for 3 days to prevent infection.

### Experimental Groups and Interventions

Twelve rats were randomly assigned to three groups: SCI rats (*n* = 4), chitosan-treated SCI rats (*n* = 4), chitosan-hydroxyapatite-treated SCI rats (*n* = 4). 15 mg/kg concentrations of chitosan-hydroxyapatite and chitosan were intravenously administered daily for 5 days and started at 1 h after injury. After injury, the mice in the SCI group were injected with saline solution through the tail vein. The other groups were administrated with 15 mg/kg concentration of chitosan-hydroxyapatite, 15 mg/kg concentration of chitosan (500 μL in saline) through a single intravenous tail vein injection.

### Cy5.5-Labeled Chitosan-Hydroxyapatite Nanoparticles Treatment

Cy5.5 was dissolved in DMSO and added to chitosan or chitosan-hydroxyapatite solution for 6 h at room temperature in the dark. The solution was performed with dialysis against distilled water. The content of Cy5.5 in the labeled chitosan or chitosan-hydroxyapatite was determined using fluorescence method to ensure that the two samples had the same labeled density and were comparable. Briefly, 15 mg/kg concentrations of Cy5.5 labeled chitosan-hydroxyapatite and chitosan nanoparticles were intravenously administered in SCI rats at 1 h post-injury, respectively. At 48 h after the injection, the rats were sacrificed and the tissues of injured spinal cord were harvested. The cell nucleus was stained with 4′,6-diamidino-2-phenylindole (DAPI), respectively. The collected spinal cord tissue was placed on a slide, stained with 15 μLDAPI, and observed after 15 min. The fluorescence images of Cy5.5 labeled to chitosan-hydroxyapatite and chitosan nanoparticles in the injured spinal cord were captured with a laser confocal microscope (Leica).

### Behavioral Assessment

The locomotor activity was assessed at 1, 3, 7, 14, 21, 28 days post-injury using the Basso Beattie Bresnahan (BBB) locomotor score method. The rats were put into an open box, and the wall of the box was tapping to make them crawl. The movement distance, hip, knee and ankle walking, trunk movement and coordination of the animals were observed. Each rat was observed for 15 min, and the movement ability of the rats in each group was tested by BBB score. The final score for each animal was obtained by averaging values from both investigators.

### HE Staining

The cavity volume and radiated structure were evaluated by HE staining. After the last injection, the rats were anesthetized to death, and 2 cm of damaged spinal cord was extracted, and the spinal cord was treated with phosphate buffer (PBS) washing. The 5 μm longitudinal sections were made from the paraffin embedded blocks and stained with hematoxylin solution for 5 min. Then the sections were stained with eosin solution for 3 min and followed by dehydration with graded alcohol and clearing in xylene. The mounted slides were then observed and photographed using a light microscope (Nikon, Tokyo, Japan).

### Statistical Analysis

The data are expressed as the means ± standard deviations of three independent experiments. The statistical differences were calculated by the Student's *t*-test or one-way ANOVA analysis of variance with Dunnett's test. The data were considered statistically significant when values reached *P* < 0.05.

## Data Availability Statement

The raw data supporting the conclusions of this article will be made available by the authors, without undue reservation.

## Ethics Statement

The animal study was reviewed and approved by Shanghai Ouean University.

## Author Contributions

C-cM: conceptualization, methodology, validation, formal analysis, investigation, writing—original draft, writing—review and editing, visualization, and supervision. X-cW: conceptualization, methodology, resources, supervision, and project administration. N-pT: conceptualization, methodology, resources, supervision, project administration, and funding acquisition. All authors contributed to the article and approved the submitted version.

## Conflict of Interest

The authors declare that the research was conducted in the absence of any commercial or financial relationships that could be construed as a potential conflict of interest.

## Publisher's Note

All claims expressed in this article are solely those of the authors and do not necessarily represent those of their affiliated organizations, or those of the publisher, the editors and the reviewers. Any product that may be evaluated in this article, or claim that may be made by its manufacturer, is not guaranteed or endorsed by the publisher.
